# Notes and News

**Published:** 1892-12-24

**Authors:** 


### NOTES AND NEWS.

COLLECTED AND COMMUNICATED.

WE must heartily congratulate Mr. George P. Field, the chairman of the extension committee; Mr. Thomas Ryan, the secretary, and all who were charged with the arrangements for laying the foundation-stone of the Clarence Memorial wing of St. Mary's Hospital, on Saturday last. Their Royal Highnesses the Prince of Wales, the Duke of York, Princess Maud, and the Duke of Cambridge showed their gratification, and it was certainly well deserved. We doubt if a similar ceremonial has ever been so well organised, and we are confident it never gave greater content to a large audience and the immediate participants. Indeed, we are glad to hear that the Duke of Cambridge said, after the proceedings concluded, that it was the most interesting, and by far the best managed ceremony he had ever been present at. Wealthy people and others who wish to expend their Christmas offerings wisely cannot do better than send a cheque to the Secretary of St. Mary's Hospital, Paddington, for the Clarence Memorial Fund.

THERE is an excellent little institution—the only one of its kind—which is not as generally known as it should be. We allude to the After Care Association for Poor Female Convalescents on leaving Asylums for the Insane. The headquarters of the Association are at the Church House, but it is gradually establishing direct communication with, and interest in, all the larger asylums of the United Kingdom. The objects of the society are so undoubtedly useful and needful, that we feel sure many of our readers will be glad to learn that so good a work is being carried on. The annual report of the Association contains the record of cases where assistance given has been the means of securing competency to many who otherwise would have been cast adrift helpless on leaving the asylum. It is proposed to extend the work further by the opening of a small cottage home where patients may recruit before returning to work, when necessary. This would undoubtedly be a most beneficial arrangement, and as only £100 is required to commence the scheme, we hope shortly to hear of its accomplishment.

THE Continental Importation Society are to hold an international exhibition of hygiene, pharmaceutics, and food at the Royal Aquarium, Westminster, in January and February next.

A COMMITTEE has been formed to lay before the Legislature of New York a project for imposing restrictions on marriage. Dr. William Fiske is the chairman. The theory of hereditary tendency being granted, it is argued that the public welfare is endangered by the marriage of those tainted by insanity, dipsomaniacs, and criminals. Youthful marriages are also condemned. It will be interesting to learn how Americans propose to legislate on this difficult question.

THE Treasurer and Governors of Guy's Hospital have appointed, as Dr. Steele's successor, Dr. Edwin C. Perry, M.A., M.B. (Cantab), Fellow of King's College, Cambridge, one of the Assistant Physicians, Dean and Warden of Guy's Hospital. Dr. Perry will, we understand, retain the office of Assistant Physician, but relinquish those of Dean and Warden. Dr. Perry has had no practical experience of hospital administration, except such as he gained as house surgeon and house physician at the London Hospital during his twelve months residence there. Dr. Perry has, however, shown himself to be a man of character and ability, who has earned the goodwill of his colleagues at Guy's by successful and continuous hard work. We wish Dr. Perry a long and useful career as Medical Superintendent of Guy's Hospital, an appointment which will afford him almost unlimited opportunity for wisely exercising the great administrative gifts with which he is credited by those who know him best.

CHOLERA and leprosy are two diseases which have elicited much public concern of late. One consoling thought in the contemplation of such terrible subjects is the knowledge thus brought to light of the many earnest and self-sacrificing workers who are quietly labouring in the battle-field of disease. Numbers doubtless go to rest unknown save in the immediate circle which has benefited by their good deeds, and whose care left no time for the worker to take the hand from the plough to seek the reward of fame. But, fortunately for the encouragement and emulation of others, we become possessed of some noble records. It is with pleasure that we heard lately from the friends of two American ladies respecting their admirable examples in the care of the sufferers both from cholera and leprosy. Miss Mary Reed, a member of the Women's Foreign Missionary Society, who has consecrated her life to work amongst the lepers of India, has caught the terrible disease. Nothing daunted, she is encouraged by the fact that the disease makes little progress in her case. She has now determined to take up her permanent residence in the leper hospital at Chandag, and there continue her work. The other lady we refer to is Dr. Mary Bradford, a Presbyterian missionary, who tended nearly all the Christians suffering from cholera in Tabouz, in Persia, during the outbreak of that disease. She was the only physician in the city; some idea may, therefore, be formed of the amount of self-sacrificing labour this entailed. Except where the enthusiasm of admiring friends will not have it so, such examples are frequently never brought to public notice, being the outcome of a quiet and earnest sense of duty and Christian love.

